# Influence of electrical part of traction transmission on dynamic characteristics of railway vehicles based on electromechanical coupling model

**DOI:** 10.1038/s41598-021-97650-4

**Published:** 2021-09-15

**Authors:** Xun Wang, Tiefeng Peng, Pingbo Wu, Litong Cui

**Affiliations:** 1 State Key Laboratory of Traction Power, Southwest Jiaotong University, Chengdu, Sichuan China; 2 Shenzhen Possibler Technology Co., Ltd, Shenzhen, China

**Keywords:** Mechanical engineering, Electrical and electronic engineering

## Abstract

With the continuous development of rail transit industry and the acceleration of train speed, higher requirements are established for the operation quality of high-speed trains and the reliability of transmission system. In the process of train running, speed fluctuation and vibrations from various parts of the driving devices are common, which could be greatly affected by the traction torque. During traction transmission, the harmonic vibration torque exists in traction motor due to that the motor is connected with non-sinusoidal alternating current. In order to study the vibration influence of the electrical component of traction transmission system on the rail vehicles, i.e., bogie and car-body, an electro-mechanical coupling dynamic model for rail transit vehicles was established by explicitly incorporating the electric-induced traction into the transmission model. The dynamics responses of the vertical, lateral and longitudinal acceleration on vehicle components, such as axle box and car-body were quantitative analyzed. By comparison with field test, it was observed that there was a vibration peak of 12-times of the fundamental rotor frequency on the bogie frame and axle box, which existed at conditions of traction, uniform speed and braking. However, the vibration acceleration exhibit nearly little difference with or without traction force, especially at low frequency domain < 100 Hz.

## Introduction

**Figure 1:**
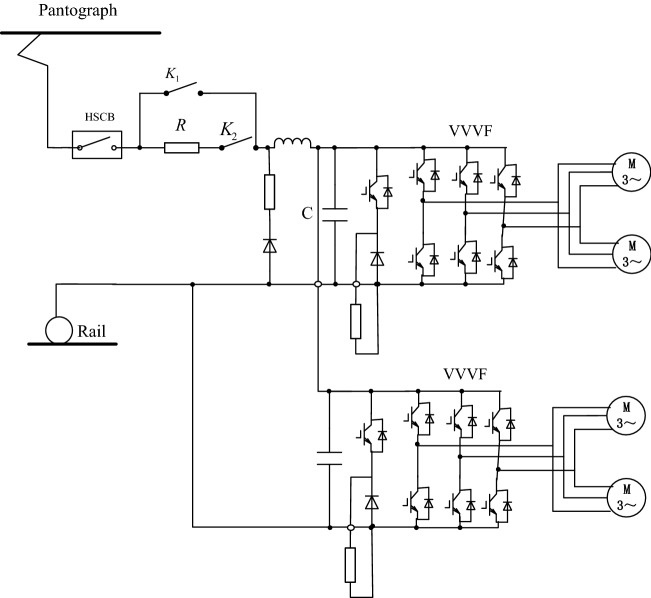
Schematic diagram of electrification for metro traction drive system.

Electric traction, which has the characteristics of less energy consumption per unit transportation volume^1^, less environmental pollution, comfort, safety, convenience and rapidity, is widely adopted in rail transit . With the rapid development of national economy, railway transportation^2,3^ plays an important role in social and economic life in China^4^, and subway^5^ has become an effective way to relieve the pressure of urban traffic. AC driving technology, which has the advantages of high speed, large power, good traction and braking characteristics, high adhesion utilization rate and high power factor, controls train operation^6,7^ by changing the torque and speed of AC asynchronous motor^8^. As one of the core components to control train operation^9^, metro traction drive system is mainly composed of intermediate DC link, traction inverter, traction motor, gear drive system, and so on. Traction drive system adopts the frame^10^ control mode, a traction inverter supplies power to two traction motors on a bogie^11^, as shown in Fig. 1.

An improved closed-loop torque and current pulsation suppression method was proposed by Wang et al.^12^, which could eliminate the *q*-axis current pulsation component in the field-oriented control system through frequency compensation. The torque pulsation suppression was also achieved automatically. Chen et al.^13^ proposed an advanced synchronous SVPWM over-modulation method, which can greatly simplify the entire modulation strategy. And they analyzed the harmonics of the advanced synchronous SVPWM over-modulation strategy and compared it with that of SHE-PWM. A simplified power system model containing regenerative inverters and trains has been built by Zhang et al*.*^14^. The impact of operating characteristics on the amount of regenerative braking energy^15^ and its distribution was analyzed. The inverter operating characteristics were optimized by a cost function considering total energy consumption, brake shoes wear, and inverter expense. Hao et al.^16^ established steady-state equivalent models of traction power systems including reversible converters, and presented the AC/DC sequential power flow algorithm based on Newton–Raphson method.

Field measurements on the vibrating characteristics of the car body (CB) and its suspended equipment (CBSE) were studied by Wu et al*.*^17^ for a high-speed railway vehicle, in their long-term tracking test, the running stability of vehicle and wheel-rail^18^ interaction were also examined with the increase of operation distance (OD), a total of 2,400,000 km. Based on the theory of structural dynamics^19^ and the principle of modal superposition method, the formula for calculating the dynamic stress of vibration fatigue^20,21^ was deduced by Wu et al. under non-stationary state. The complex frequency response function (FRF) of each mode was calculated by finite element program, and the impulse response function (IRF) of each mode was obtained by inverse fast Fourier transform (IFFT) method, so as to realize the decoupling of each mode.

Rezvani et al.^22^ focused on the bogie-carbody nonlinear dynamic interaction of a Shinkansen high-speed rail vehicle, and the Euler–Bernoulli beam model was used to simulate vertical elastic vibrations of the car-body. Shi et al.^23^ found the vehicle system vibrates at around 2.5 Hz in the lateral direction, which leads to the low-frequency swaying on the car-body. Lei et al.^24^ studied the wheel-rail contact^25^ and creep characteristics and the evolution law of corrugation with different wavelengths, where the wave-like wear^26^ was idealized as continuous harmonic excitations consisting of three wavelengths and wave depths.

Generally, the traction drive system regulates the train speed and traction by controlling the amplitude and frequency of the three-phase AC^27^, which are inputted to the traction motor. With the increase of the running speed of rail vehicles, the influence of the electrical part of traction transmission system in terms of harmonic component on the vehicle system dynamics^28^ cannot be ignored. In practice, when the inverter supplies power to the traction motor, the voltage or current obtained by the motor may contain large amount of harmonic components in addition to the fundamental wave. The non-sinusoidal AC input from traction inverter to the traction motor can cause motor vibration^29^ and affect the dynamic quality of rail vehicle^30–32^. Conventionally, the research on the operating characteristics of the traction motor generally only considers the ideal sinusoidal voltage, or fundamental component of non-sinusoidal voltage supply. Dynamics response of vehicle system^33–36^ induced by electro-mechanical coupling^37^, especially by explicitly considering harmonic component is insufficient. In this study, an electro-mechanical coupling dynamic model explicitly incorporating the electric-induced traction into the transmission was established. Based on this model, the influence of electrical part of traction transmission system on rail vehicle system was studied. The genuine contributions of the work were as follows. An electromechanical coupling dynamic vehicle model was proposed and established, this could explicitly incorporate the electric-induced traction into transmission. Vibration accelerations on various vehicle components were quantitative analyzed from the traction drive system and compared with the field test, to compare and validate the electromechanical coupling model. Due to the characteristics of high operation density, long routing and complex line operation environment of rail vehicles in China, the vibration fatigue of traction motor hanger and gearbox box has occurred in the process of long-term service. How to reduce the vibration level of traction drive system is an urgent research topic. Previous reports or literature^33,38,39^ mainly dealt with the mechanical aspects regarding vibrating, its electrical inducted vibrating especially by explicitly incorporating the electrical-induced traction is insufficient. Based on the above background, this study provided reference support for the influence from electrical part on the vibrational characters of rail vehicles.

## Establishment of vehicle model with traction transmission system

### Mathematical model of transmission system

In order to analyze the mathematical model of traction motor, the following assumptions were applied: (1) Three-phase windings of the motor are symmetrical, and the spatial harmonic magneto-motive force due to the limited number of slots is ignored. (2) Influence of magnetic saturation and core loss is ignored. (3) Self-inductance and mutual inductance of each winding are linear. (4) Influence of temperature and frequency on motor resistance is ignored. In the two-phase arbitrary rotating coordinate system, i.e., *dq* coordinate system, the mathematical model of traction motor can be expressed as follows:

Torque equation

1 $$ T_{{e}} = \frac{3}{2}n_{{p}} \frac{{L_{{m}} }}{{\sigma L{\text{s}}L{\text{r}}}}\left| {\psi_{{\text{s}}} } \right|\left| {\psi_{{\text{r}}} } \right|\sin \theta $$

Equation of motion

2 $$ T_{{e}} = T_{{L}} + \frac{J}{{n_{{p}} }}\frac{{d_{{\upomega _{{r}} }} }}{dt} $$

where: $\psi_{{\text{s}}}$—Stator flux linkage in three phase shafting. $\psi_{{\text{r}}}$—Rotor flux linkage in three phase shafting. $L_{{s}}$—Stator self-inductance. $L_{{r}}$—Rotor self-inductance. $L_{{m}}$—Mutual inductance of two phase winding. $n_{{p}}$—Polar logarithm. $J$—Moment of inertia. $\omega_{{r}}$—Angular velocity of motor rotor. $\sigma$—The leakage inductance of the motor. $\theta$—The angle between the stator flux linkage and the rotor flux linkage.

When the motor is connected to the non-sinusoidal power supply, the time harmonic magneto-motive force will be generated in the air gap of the motor, which will generate additional harmonic torque. The harmonic torque of traction motor^8^ includes stable harmonic torque and vibration harmonic torque. When the air gap harmonic flux and harmonic rotor current have the same order, their interaction will produce stable harmonic torque. If the fundamental and harmonic waves in the air gap generate *n* rotating magnetic fields, there will be (*n* − 1) stable harmonic torques. The *k*th stable harmonic torque is

3 $$ T_{k} = \pm \frac{{mn_{{\text{p}}} }}{{2\pi f_{1} }}I_{2k}^{2} \frac{{R_{{{\text{r}}k}} }}{(k \mp 1)} $$

When the times of harmonic flux and harmonic rotor current are different, their interaction will produce vibration harmonic torque. If the fundamental and harmonic waves in the air gap generate *n* rotating magnetic fields, there will be (*n*^2^ − *n*) vibration harmonic torques. The harmonic vibration torque of the fifth harmonic is

4 $$ T_{5 - 1} = \frac{{3n_{{\text{p}}} }}{{2\pi f_{1} }}I_{25} E_{2} \cos (6\omega t - \phi_{2} ) = \frac{{3n_{{\text{p}}} }}{{2\pi f_{1} }}I_{25} E_{2} \cos (6\omega t + \pi - \phi_{2} ) $$

The harmonic vibration torque of the 7th harmonic is

5 $$ T_{7 - 1} = \frac{{3n_{{\text{p}}} }}{{2\pi f_{1} }}I_{27} E_{2} \cos (6\omega t - \phi_{2} ) $$

Similarly, the interaction between the 11th and 13th harmonic currents and the fundamental magnetic field will produce the 12th harmonic torque.

**Figure 2:**
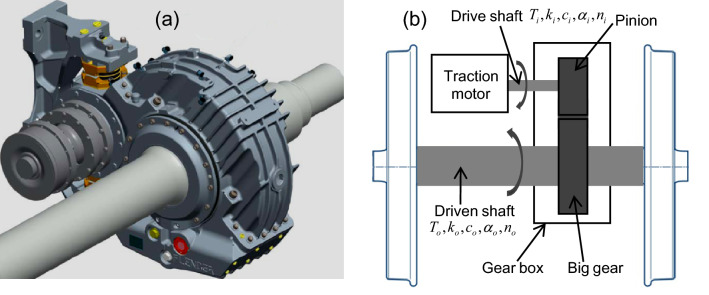
Gearbox model (a) and its sketch illustration of torsional vibration model (b) for a typical rail train.

Gear transmission system^40^ is simplified as pure torsional vibration mode, shown in Fig. 2, only the torque transfer between the driving gear and the passive gear is considered. $k_{{\text{i}}}$ and $k_{0}$ are the torsional stiffness of the driving shaft and the driven shaft, respectively, $c_{{\text{i}}}$ and $c_{0}$ are their torsional damping, $\alpha_{{\text{i}}}$ and $\alpha_{0}$ are torsional angular displacement, $n_{{\text{i}}}$ and $n_{0}$ are angular velocity, $T_{{\text{i}}}$ and $T_{0}$ are driving torque.

The gear ratio is defined as:

6 $$ N = \frac{{n_{{\text{i}}} }}{{n_{0} }} $$

Dynamic equation of the transmission torque of the gear is as follows:

7 $$ T = k\left( {\alpha_{0} - \frac{{\alpha_{{\text{i}}} }}{N}} \right) $$

Since the left and right wheels have the same rotational inertia, the torsion vibration equation of the transmission system can be obtained:

8 $$ \left[ \begin{gathered} J_{{1{\kern 1pt} {\kern 1pt} }} {\kern 1pt} {\kern 1pt} {\kern 1pt} {\kern 1pt} {\kern 1pt} {\kern 1pt} {\kern 1pt} {\kern 1pt} {\kern 1pt} {\kern 1pt} {\kern 1pt} {\kern 1pt} {\kern 1pt} 0 \hfill \\ {\kern 1pt} {\kern 1pt} {\kern 1pt} {\kern 1pt} 0{\kern 1pt} {\kern 1pt} {\kern 1pt} {\kern 1pt} {\kern 1pt} {\kern 1pt} {\kern 1pt} {\kern 1pt} {\kern 1pt} {\kern 1pt} {\kern 1pt} J_{2} \hfill \\ \end{gathered} \right]\left[ \begin{gathered} \ddot{\theta }_{1} \hfill \\ \ddot{\theta }_{2} \hfill \\ \end{gathered} \right] + \left[ \begin{gathered} {\kern 1pt} {\kern 1pt} {\kern 1pt} {\kern 1pt} {\kern 1pt} {\kern 1pt} {\kern 1pt} {\kern 1pt} {\kern 1pt} {\kern 1pt} c{\kern 1pt} {\kern 1pt} {\kern 1pt} {\kern 1pt} {\kern 1pt} {\kern 1pt} {\kern 1pt} {\kern 1pt} {\kern 1pt} {\kern 1pt} {\kern 1pt} {\kern 1pt} {\kern 1pt} {\kern 1pt} {\kern 1pt} - c_{2} \hfill \\ {\kern 1pt} - c_{1} {\kern 1pt} {\kern 1pt} {\kern 1pt} {\kern 1pt} {\kern 1pt} {\kern 1pt} {\kern 1pt} {\kern 1pt} {\kern 1pt} {\kern 1pt} {\kern 1pt} {\kern 1pt} {\kern 1pt} {\kern 1pt} 2c_{2} \hfill \\ \end{gathered} \right]\left[ \begin{gathered} \dot{\theta }_{1} \hfill \\ \dot{\theta }_{2} \hfill \\ \end{gathered} \right] + \left[ \begin{gathered} {\kern 1pt} {\kern 1pt} {\kern 1pt} {\kern 1pt} {\kern 1pt} k{\kern 1pt} {\kern 1pt} {\kern 1pt} {\kern 1pt} {\kern 1pt} {\kern 1pt} {\kern 1pt} {\kern 1pt} {\kern 1pt} {\kern 1pt} {\kern 1pt} {\kern 1pt} {\kern 1pt} {\kern 1pt} {\kern 1pt} {\kern 1pt} {\kern 1pt} {\kern 1pt} {\kern 1pt} {\kern 1pt} - k_{2} \hfill \\ - k_{1} {\kern 1pt} {\kern 1pt} {\kern 1pt} {\kern 1pt} {\kern 1pt} {\kern 1pt} {\kern 1pt} {\kern 1pt} {\kern 1pt} {\kern 1pt} {\kern 1pt} {\kern 1pt} {\kern 1pt} {\kern 1pt} {\kern 1pt} {\kern 1pt} 2k_{2} \hfill \\ \end{gathered} \right]\left[ \begin{gathered} \theta_{1} \hfill \\ \theta_{2} \hfill \\ \end{gathered} \right] = \left[ \begin{gathered} - \frac{{J_{{1{\kern 1pt} {\kern 1pt} }} }}{J}T_{{\text{e}}} - T_{1} {\kern 1pt} \hfill \\ {\kern 1pt} {\kern 1pt} {\kern 1pt} {\kern 1pt} {\kern 1pt} {\kern 1pt} {\kern 1pt} {\kern 1pt} {\kern 1pt} {\kern 1pt} {\kern 1pt} {\kern 1pt} {\kern 1pt} {\kern 1pt} {\kern 1pt} T_{1} - T_{2} {\kern 1pt} \hfill \\ \end{gathered} \right] $$

where: $J$—the equivalent rotational inertia of the whole transmission system equivalent to the wheel shaft. $J_{1}$—the moment of inertia of the left wheel. $J_{2}$—the moment of inertia of the right wheel. $k_{1}$—the equivalent torsion stiffness between the transmission system and the wheel-set. $k_{2}$—torsion stiffness of wheel-set. $c_{1}$*—*equivalent torsion damping between the transmission system and the wheel-set. $c_{2}$—torsion damping of wheel-set. $T_{{\text{e}}}$—electromagnetic torque output of traction motor. $T_{1}$—the counteracting moment of the rail to the left wheel. $T_{2}$—the counteracting moment of the rail to the right wheel. $\theta_{1}$—the torsion angle displacement between the traction motor and the left wheel. $\theta_{2}$—the torsion angle displacement between the left and right wheels.

### Control model of traction drive system

Generally, direct torque control (DTC) is to directly control the switching state of the inverter according to the comparison between the measured value and the observed value of flux and torque. In order to realize the feedback control in the direct torque control system, it is necessary to accurately estimate the current stator flux and torque. The stator flux is estimated as follows:

9 $$ \left\{ \begin{gathered} \psi_{{{\text{s}}\upalpha }} { = }\int {(u_{{{\text{s}}\upalpha }} - i_{{{\text{s}}\upalpha }} R_{{\text{s}}} )dt} \hfill \\ \psi_{{{\text{s}}\upbeta }} { = }\int {(u_{{{\text{s}}\upbeta }} - i_{{{\text{s}}\upbeta }} R_{{\text{s}}} )dt} \hfill \\ \end{gathered} \right. $$

The observed values of electromagnetic torque are as follows:

10 $$ T_{{\text{e}}} = n_{{\text{p}}} (\hat{\psi }_{{{\text{s}}\upalpha }} i_{{{\text{s}}\upbeta }} - \hat{\psi }_{{{\text{s}}\upbeta }} i_{{{\text{s}}\upalpha }} ) $$

**Figure 3:**
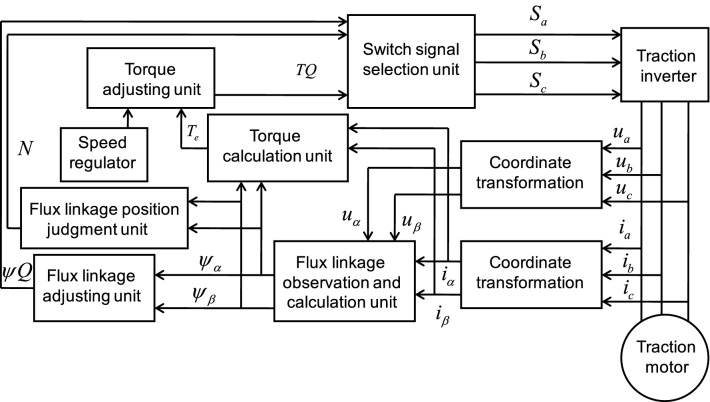
Block diagram of direct torque control for traction motor.

Direct torque control is mainly composed of several parts in Fig. 3, the output voltage and current signals of traction inverter could be achieved by measurement. Flux $\psi_{\alpha }$ and $\psi_{\beta }$ could be obtained by flux observation and calculation unit using voltage and current signals, and then, the actual torque value, which is recorded as $T_{{\text{e}}}$ could be obtained by torque calculation unit. $\psi_{\alpha }$ and $\psi_{\beta }$ would get the flux regulation signal $\psi Q$ through the flux linkage adjusting unit. At the same time, the interval number of the flux linkage can be calculated by the flux linkage position judgment unit. Through the torque adjusting unit, the torque adjusting signal $TQ$ can be obtained. $\psi Q$, $TQ$ and *N*, at the same time, are sent to the switch signal selection unit as inputs to confirm the current voltage vector, and output the voltage switch signal to the traction inverter. So the self-control of torque and flux could be realized. The torque adjusting unit and the flux linkage adjusting unit generally use the Schmidt trigger to form the hysteresis comparator.

In the voltage source inverter, the switch states of the same group are always opposite. If one phase of the three-phase load is connected with the positive pole of the DC power supply, the corresponding switch state is 1. Otherwise, when it is connected with the negative pole of the DC power supply, the corresponding switch state is 0. So, there are eight types of switch states, which are as follows: *U*_0_ (000), *U*_1_ (001), *U*_2_ (010), *U*_3_ (011), *U*_4_ (100), *U*_5_ (101), *U*_6_ (110), *U*_7_ (111).

### Vehicle system dynamics model

**Figure 4:**
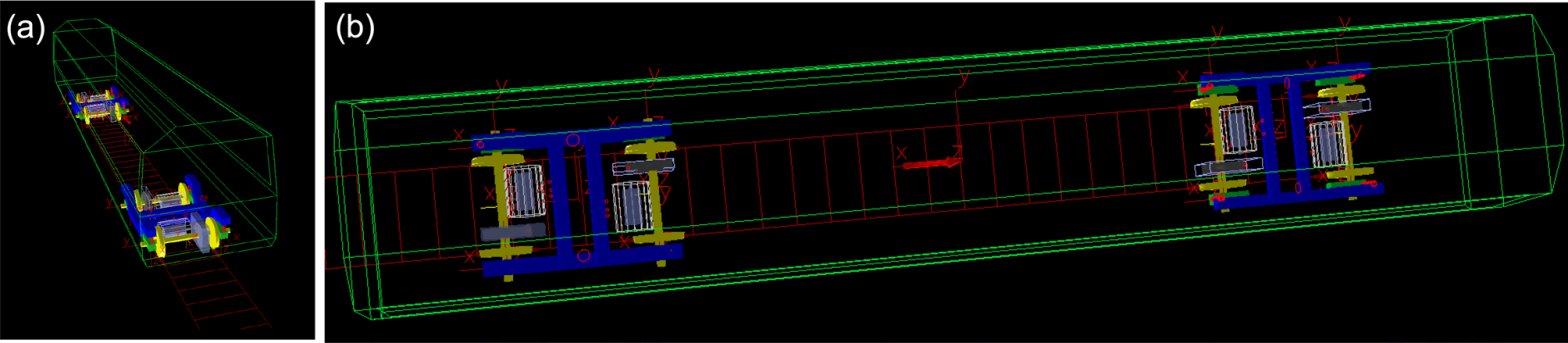
Side view (a) and top view (b) of the vehicle dynamics model with transmission system.

The multi-body dynamic model of rail transit vehicle was established and shown in Fig. 4. It included a car-body, two bogie frames, four wheel sets^41,42^ and eight axle boxes, and four transmission sets. Each drive system consists of a traction motor, a motor hanger, a coupling and a gearbox. The matrix form of the motion differential equation of the whole vehicle system is

11 $$ \left[ M \right]\left\{ {\ddot{x}} \right\} + \left[ C \right]\left\{ {\dot{x}} \right\} + \left[ K \right]\left\{ x \right\} = \left\{ F \right\} $$

where: $\left[ M \right]$—The quality matrix of the system. $\left[ C \right]$—System damping matrix. $\left[ K \right]$—System stiffness matrix. $\left\{ x \right\}$—System displacement vector. $\left\{ {\dot{x}} \right\}$—System velocity vector. $\left\{ {\ddot{x}} \right\}$—System acceleration vector. $\left\{ F \right\}$—The external force vector of the system.

Traction motor was fixed on the motor hanger through bolt connection^43^. Passive gear end of the gearbox box was fixed on the wheel-set shaft^44^ through bearing, and driving gear end was connected with the bogie frame through the suspension device. Only the degree of freedom of rotation around Y-axis of the coordinate system was released between the box and the wheel-set shaft. Passive gear and the wheel-set shaft were fixed, and they could rotate synchronously around the Y-axis of the coordinate system. The driving gear and the traction motor rotor were connected through a coupling, in which the coupling was realized by constraining the speed synchronization of the rotor and driving gear.

The coupling equation of the electro-mechanical system is expressed as follows

12 $$ \left[ \begin{gathered} J_{{1{\kern 1pt} {\kern 1pt} }} {\kern 1pt} {\kern 1pt} {\kern 1pt} {\kern 1pt} {\kern 1pt} {\kern 1pt} {\kern 1pt} {\kern 1pt} {\kern 1pt} {\kern 1pt} {\kern 1pt} {\kern 1pt} {\kern 1pt} 0 \hfill \\ {\kern 1pt} {\kern 1pt} {\kern 1pt} {\kern 1pt} 0{\kern 1pt} {\kern 1pt} {\kern 1pt} {\kern 1pt} {\kern 1pt} {\kern 1pt} {\kern 1pt} {\kern 1pt} {\kern 1pt} {\kern 1pt} {\kern 1pt} J_{2} \hfill \\ \end{gathered} \right]\left[ \begin{gathered} \ddot{\theta }_{1} \hfill \\ \ddot{\theta }_{2} \hfill \\ \end{gathered} \right] + \left[ \begin{gathered} {\kern 1pt} {\kern 1pt} {\kern 1pt} {\kern 1pt} {\kern 1pt} {\kern 1pt} {\kern 1pt} {\kern 1pt} {\kern 1pt} {\kern 1pt} c{\kern 1pt} {\kern 1pt} {\kern 1pt} {\kern 1pt} {\kern 1pt} {\kern 1pt} {\kern 1pt} {\kern 1pt} {\kern 1pt} {\kern 1pt} {\kern 1pt} {\kern 1pt} {\kern 1pt} {\kern 1pt} {\kern 1pt} - c_{2} \hfill \\ {\kern 1pt} - c_{1} {\kern 1pt} {\kern 1pt} {\kern 1pt} {\kern 1pt} {\kern 1pt} {\kern 1pt} {\kern 1pt} {\kern 1pt} {\kern 1pt} {\kern 1pt} {\kern 1pt} {\kern 1pt} {\kern 1pt} {\kern 1pt} 2c_{2} \hfill \\ \end{gathered} \right]\left[ \begin{gathered} \dot{\theta }_{1} \hfill \\ \dot{\theta }_{2} \hfill \\ \end{gathered} \right] + \left[ \begin{gathered} {\kern 1pt} {\kern 1pt} {\kern 1pt} {\kern 1pt} {\kern 1pt} k{\kern 1pt} {\kern 1pt} {\kern 1pt} {\kern 1pt} {\kern 1pt} {\kern 1pt} {\kern 1pt} {\kern 1pt} {\kern 1pt} {\kern 1pt} {\kern 1pt} {\kern 1pt} {\kern 1pt} {\kern 1pt} {\kern 1pt} {\kern 1pt} {\kern 1pt} {\kern 1pt} {\kern 1pt} {\kern 1pt} - k_{2} \hfill \\ - k_{1} {\kern 1pt} {\kern 1pt} {\kern 1pt} {\kern 1pt} {\kern 1pt} {\kern 1pt} {\kern 1pt} {\kern 1pt} {\kern 1pt} {\kern 1pt} {\kern 1pt} {\kern 1pt} {\kern 1pt} {\kern 1pt} {\kern 1pt} {\kern 1pt} 2k_{2} \hfill \\ \end{gathered} \right]\left[ \begin{gathered} \theta_{1} \hfill \\ \theta_{2} \hfill \\ \end{gathered} \right] = \left[ \begin{gathered} - \frac{{J_{{1{\kern 1pt} {\kern 1pt} }} }}{J}\left( {T_{L} + \frac{J}{{n_{{\text{p}}} }}\frac{{d\dot{\theta }_{1} }}{dt} + \frac{D}{{n_{{\text{p}}} }}\dot{\theta }_{1} } \right) - T_{1} {\kern 1pt} \hfill \\ {\kern 1pt} {\kern 1pt} {\kern 1pt} {\kern 1pt} {\kern 1pt} {\kern 1pt} {\kern 1pt} {\kern 1pt} {\kern 1pt} {\kern 1pt} {\kern 1pt} {\kern 1pt} {\kern 1pt} {\kern 1pt} {\kern 1pt} {\kern 1pt} {\kern 1pt} {\kern 1pt} {\kern 1pt} {\kern 1pt} {\kern 1pt} {\kern 1pt} {\kern 1pt} {\kern 1pt} {\kern 1pt} {\kern 1pt} {\kern 1pt} {\kern 1pt} {\kern 1pt} {\kern 1pt} {\kern 1pt} {\kern 1pt} {\kern 1pt} {\kern 1pt} {\kern 1pt} {\kern 1pt} {\kern 1pt} {\kern 1pt} {\kern 1pt} {\kern 1pt} {\kern 1pt} {\kern 1pt} {\kern 1pt} {\kern 1pt} {\kern 1pt} {\kern 1pt} {\kern 1pt} {\kern 1pt} {\kern 1pt} {\kern 1pt} {\kern 1pt} {\kern 1pt} {\kern 1pt} {\kern 1pt} {\kern 1pt} {\kern 1pt} {\kern 1pt} {\kern 1pt} {\kern 1pt} {\kern 1pt} {\kern 1pt} {\kern 1pt} {\kern 1pt} {\kern 1pt} T_{1} - T_{2} {\kern 1pt} \hfill \\ \end{gathered} \right] $$

In electro-mechanical coupling model simulation, given an expected motor rotor speed, the output current motor rotor speed would be compared with it. If the expected value is larger, the output electromagnetic torque is positive and motor rotor speed will be larger. On the contrary, the motor rotor speed would be decreased. If the two values are equal, the output electromagnetic torque is motor load torque. In the multi-body dynamics vehicle model within SIMPACK package, the dynamic responses of the traction system could be identified from vehicle components, such as frame, car body and axle box. The traction torque could be obtained by direct torque control model, which could be applied to the motor rotor of vehicle model to realize the power transmission from the motor rotor to the wheel-set. Some key information of the vehicle parameters and input conditions were provided in “Appendix I”, the parameters of traction motor and control model were in “Appendix II”.

## Results and discussion

Using the electro-mechanical coupling simulation model, and comparing with the line test data, the influence of the traction system on the frame and car-body was studied.

**Figure 5:**
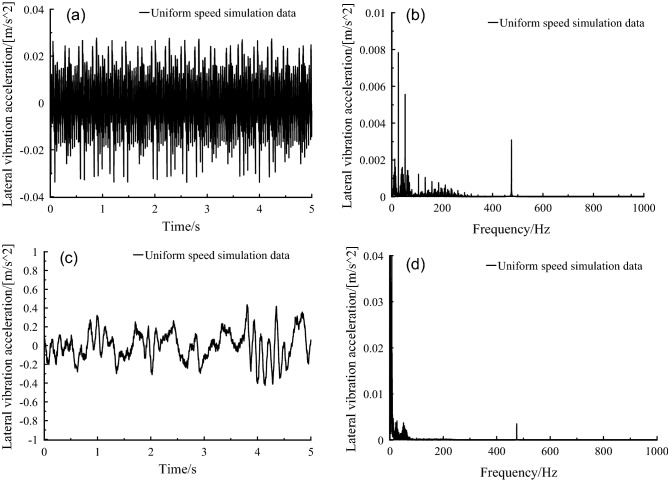
Frame lateral vibration acceleration, (a-b) orbit free spectrum, (c-d) orbital spectrum.

Frequency domain diagram of frame lateral vibration under uniform speed condition is shown in Fig. 5, a vibration peak of high frequency was very significant, i.e., 473 Hz, which was 12-times of the fundamental frequency of the motor rotor. The vibration harmonic torque, which was generated by the interaction between the 11th and 13th harmonic currents and the fundamental flux, caused the vibration frequency peak at 473 Hz.

**Figure 6:**
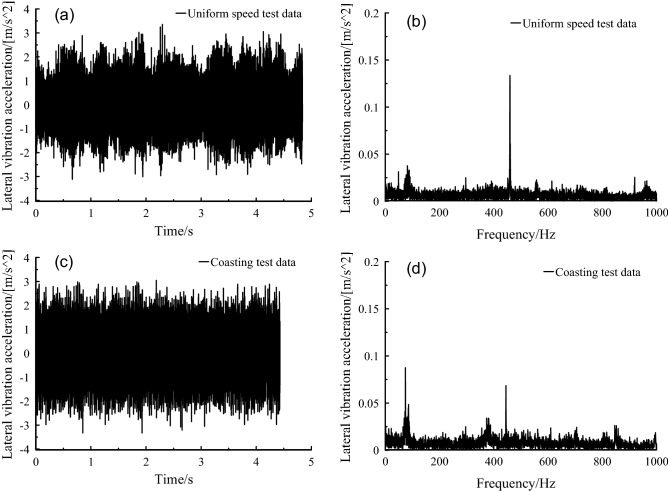
Frame lateral vibration acceleration, at (a-b) uniform speed, and (c-d) coasting condition.

The vibration frequency 469 Hz, that is, 12-times the fundamental frequency of the rotor, was also impressive under the uniform speed condition in Fig. 6, and its amplitude decreased under the coasting condition. It indicated that the lateral vibration at frame caused by the harmonic vibration torque could not disappear quickly after the power was cut off.

**Figure 7:**
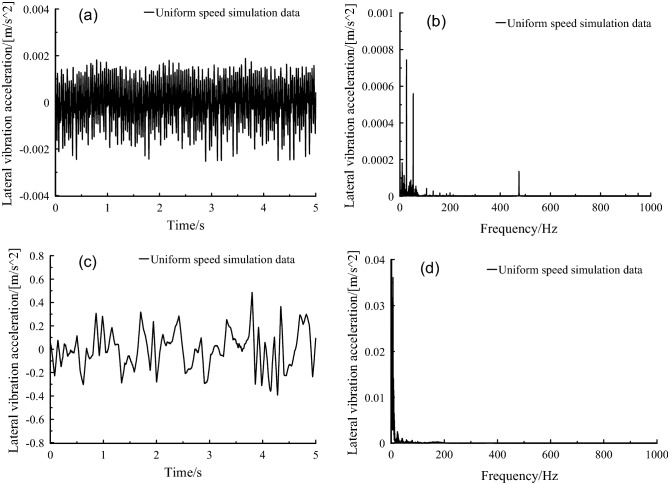
Axle box lateral vibration acceleration, (a-b) orbit free spectrum, (c-d) orbital spectrum.

It could be found from the spectrum in Fig. 7 that the frequency was mainly concentrated below 100 Hz. In the case of no orbit spectrum, 12-times of the fundamental frequency of the rotor could be identified, which located at frequency peak ~ 470 Hz. When the orbit spectrum was introduced, the low frequency vibration amplitude increased, but its peak ~ 470 Hz nearly disappeared.

**Figure 8:**
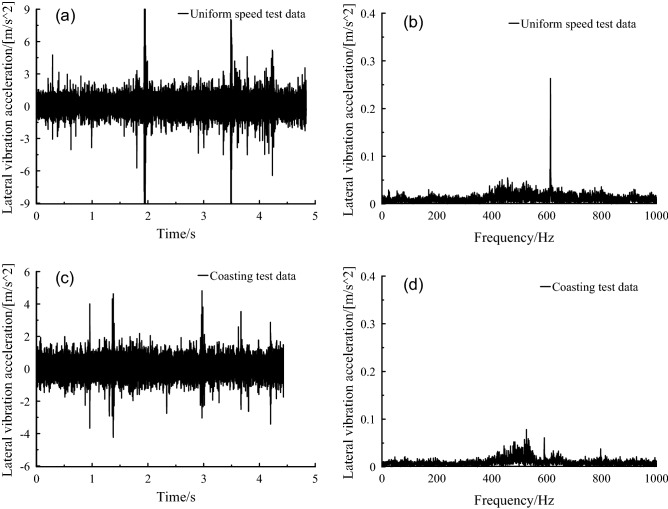
Axle box lateral vibration acceleration, at (a-b) uniform speed, (c-d) coasting condition.

An impressive frequency peak with 628 Hz could be observed from Fig. 8b, that is, meshing frequency, which was not prominent under the coasting condition. It indicated that the influence of meshing frequency on the lateral vibration of axle box was greatly reduced after the power supply was cut off. The frequency distribution was basically the same under uniform speed and coasting condition.

**Figure 9:**
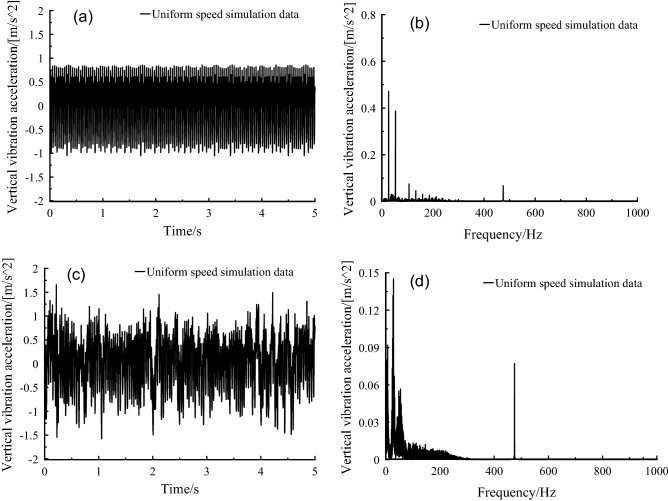
Frame vertical vibration acceleration, (a-b) orbit free spectrum, (c-d) orbital spectrum.

The vibration with orbit spectrum was slightly higher than that without orbit spectrum, as shown in the time domain diagram of Fig. 9. While, in the frequency domain diagram, the vibration frequencies of the two cases were mainly concentrated below 200 Hz, and there were two enhanced vibrations between 20 and 50 Hz. In the high frequency band of orbit free spectrum and orbital spectrum, 12-times of the fundamental frequency of the rotor appeared, that is, 473 Hz.

**Figure 10:**
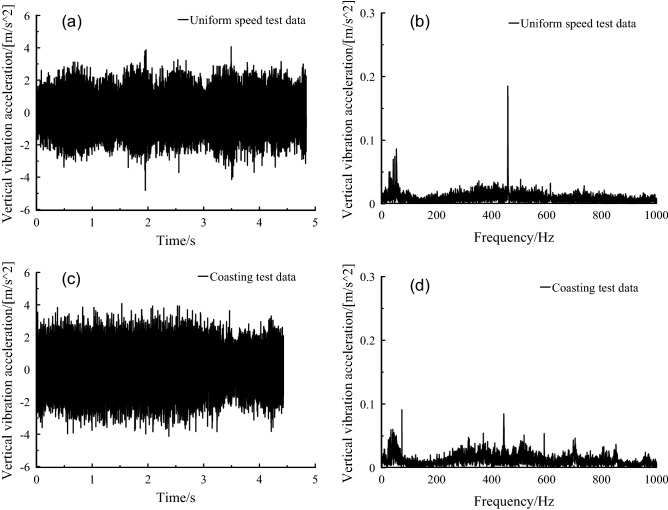
Frame vertical vibration acceleration, at (a-b) uniform speed, (c-d) coasting condition.

From the time domain diagram in Fig. 10, under the condition of constant speed and coasting, there was little difference in the amplitude of vibration acceleration. It could be seen that in the 0–1000 Hz range, most of the frequency amplitudes were below 0.05 in the frequency domain diagram of Fig. 10. The frequency 469 Hz was more prominent at high frequency, which was 12-times of the fundamental frequency of the rotor, and its amplitude in coasting condition was significantly smaller than that under the uniform speed condition.

**Figure 11:**
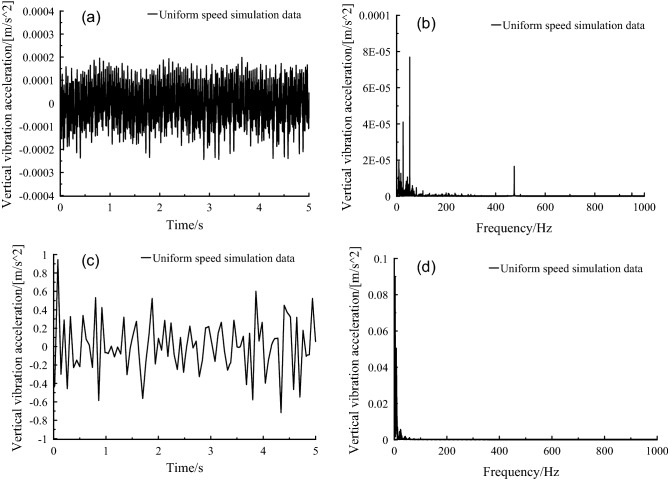
Axle box vertical vibration acceleration, (a-b) orbit free spectrum, (c-d) orbital spectrum.

Compared with orbit-free spectrum, in the time domain diagram (Fig. 11), vibration amplitude with the orbital spectrum increased significantly. In the frequency domain diagram of Fig. 11, the frequency amplitude without orbit spectrum was very small and concentrated below 100 Hz. However, the frequency amplitude with the orbit spectrum increased significantly and concentrated below 50 Hz.

**Figure 12:**
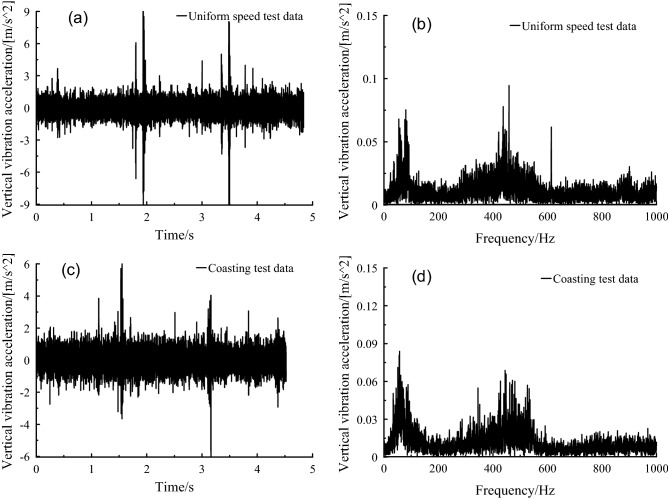
Axle box vertical vibration acceleration, at (a-b) uniform speed, (c-d) coasting condition.

The frequency distribution range of vertical vibration of axle box was basically the same under uniform speed and coasting conditions in the frequency domain diagram of Fig. 12, and the enhancement vibration occurred at 60–90 Hz and 430–540 Hz. Compared with the coasting condition, the vibration peak value of the meshing frequency, i.e. 628 Hz, was more significant under the uniform speed condition. This indicated that the influence of the meshing frequency on the vertical direction of the axle box was greatly reduced after the power supply was cut off.

**Figure 13:**
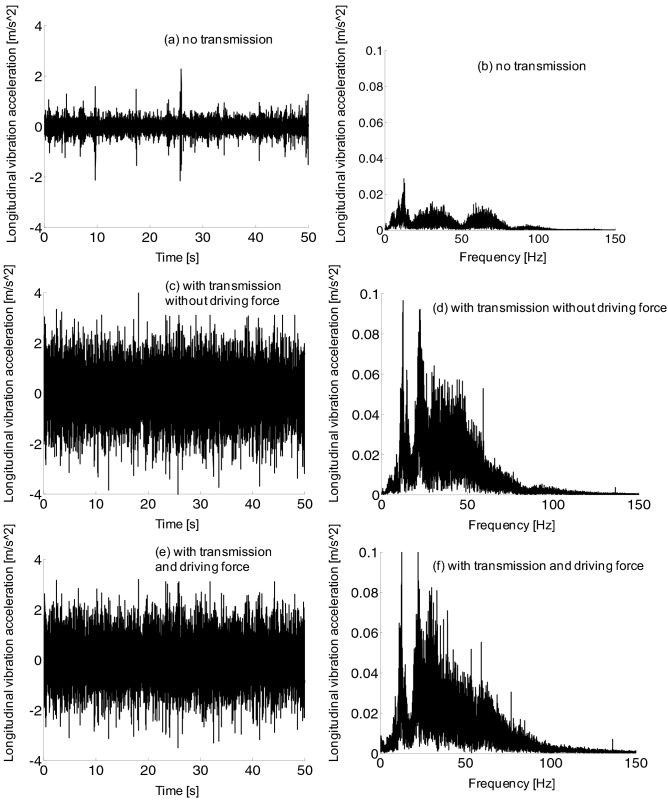
Frame longitudinal vibration acceleration, (a-b) no transmission, (c-d) with transmission without driving force, (e–f) with transmission and driving force.

From the frequency domain diagram in Fig. 13, the longitudinal vibration frequency of the frame is distributed between 0 and 100 Hz, and there is a large vibration amplitude between 0 and 70 Hz. Compared with the waveform without transmission device, the amplitude of longitudinal vibration acceleration of the frame with transmission device increased. However, compared the vibration acceleration with and without traction force, there was little difference on longitudinal vibration frequency of the frame.

**Figure 14:**
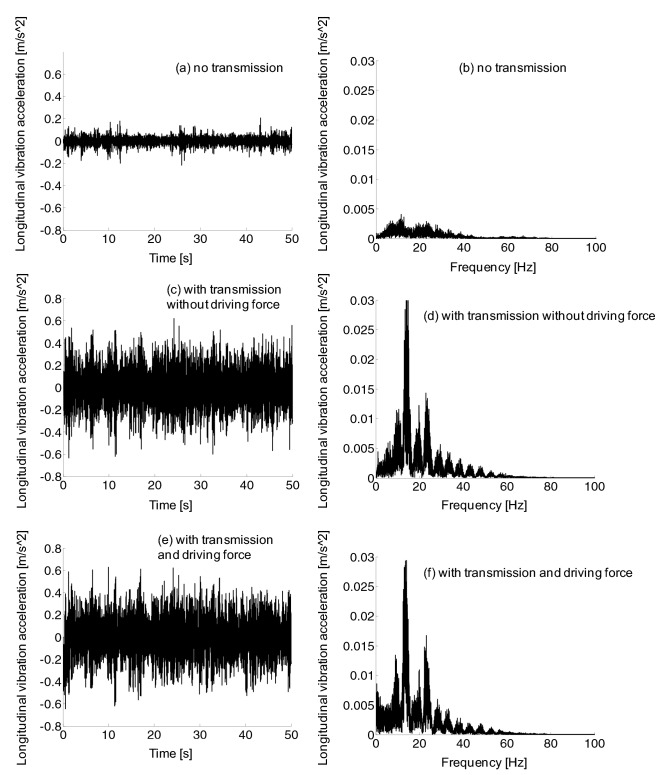
Car body longitudinal vibration acceleration, no transmission (a-b), with transmission without driving force (c-d), and with transmission and driving force (e–f).

No matter the frequency domain diagram or the time domain diagram, in Fig. 14, longitudinal vibration amplitude of the car body without transmission system was much lower than that of the car body with transmission system. Meanwhile, the frequency range of longitudinal vibration of the car body with or without traction force was roughly the same at 0–60 Hz, and vibration amplitude strengthened between 10 and 25 Hz.

**Figure 15:**
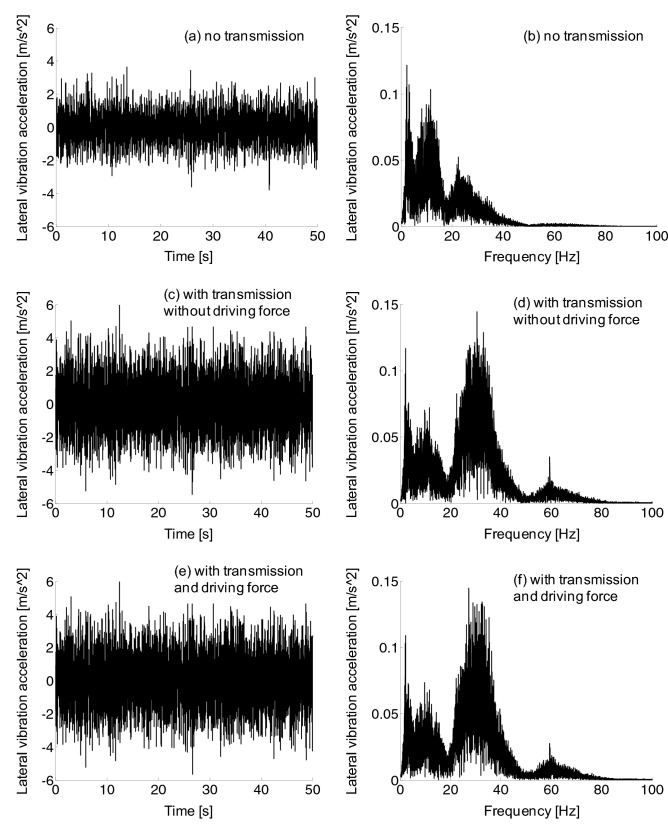
Frame lateral vibration acceleration, (a-b) no transmission, (c-d) with transmission without driving force, (e–f) with transmission and driving force.

In the frequency domain diagram of frame lateral vibration of Fig. 15, the frequency was mainly distributed in 0–80 Hz. Compared with the non-transmission system, the lateral vibration amplitude of the frame with transmission system increased substantially in the frequency domain of 20–40 Hz and 50–70 Hz. However, the driving force had little effect on the lateral vibration of the frame.

**Figure 16:**
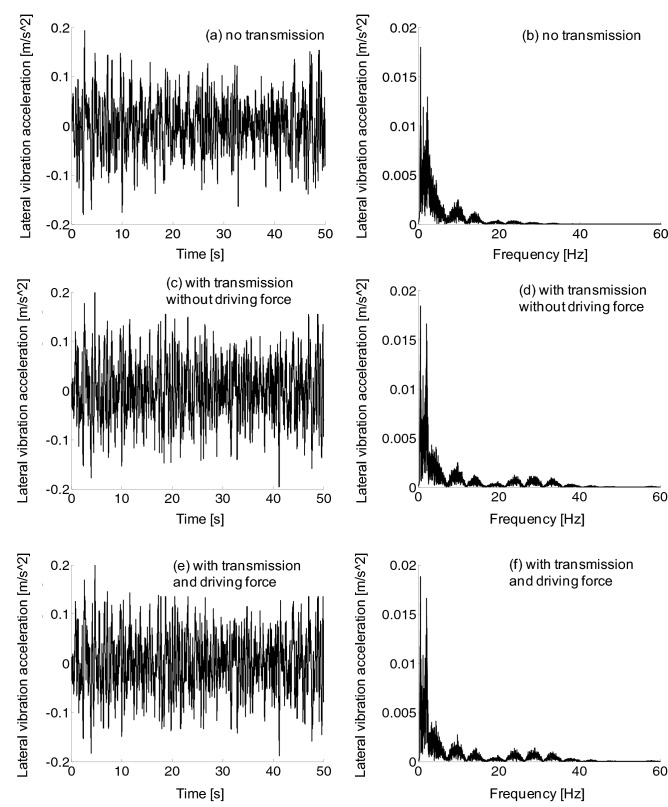
Car body lateral vibration acceleration, (a-b) no transmission, (c-d) with transmission without driving force, (e–f) with transmission and driving force.

The lateral vibration frequency of the car body was mainly distributed in 0–7 Hz, as shown in Fig. 16, and the vibration amplitude was below 0.02 m/s^2. However, the lateral vibration acceleration of car body exhibited nearly little difference with or without traction force.

**Figure 17:**
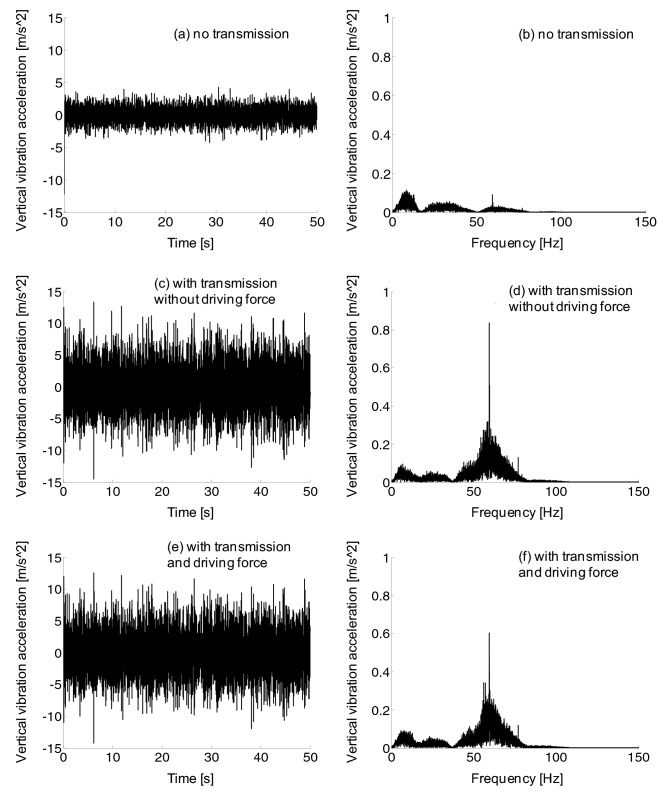
Frame vertical vibration acceleration, (a-b) no transmission, (c-d) with transmission without driving force, (e–f) with transmission and driving force.

The vertical vibration frequency of the frame was mainly distributed in 0–100 Hz in frequency domain diagram of Fig. 17, compared with the non-transmission system, and the vertical vibration amplitude of the frame with transmission system at 50–70 Hz was significantly enhanced. In time domain diagram of Fig. 17, the vibration amplitude of frame without transmission system was significantly higher than that of frame with transmission system.

**Figure 18:**
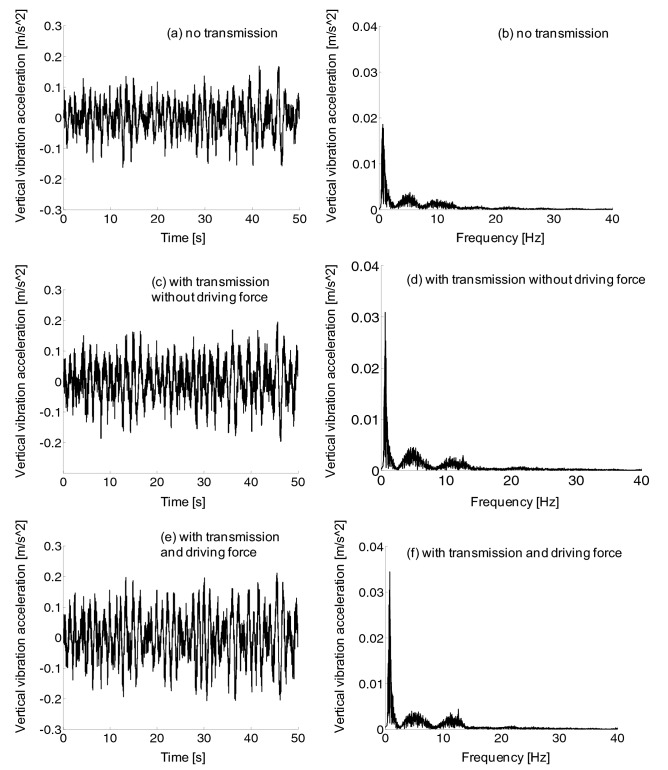
Car body vertical vibration acceleration, (a-b) no transmission, (c-d) with transmission without driving force, (e–f) with transmission and driving force.

It can be seen from the frequency domain diagram in Fig. 18 that the vertical vibration frequency distribution of the car body was in the range of 0–15 Hz, with 0–2 Hz as the main frequency distribution area. Through the second suspension system, the vibration amplitude was significantly suppressed, that is, from frame to car-body, which could be validated from the comparison of Figs. 17 and 18.

**Figure 19:**
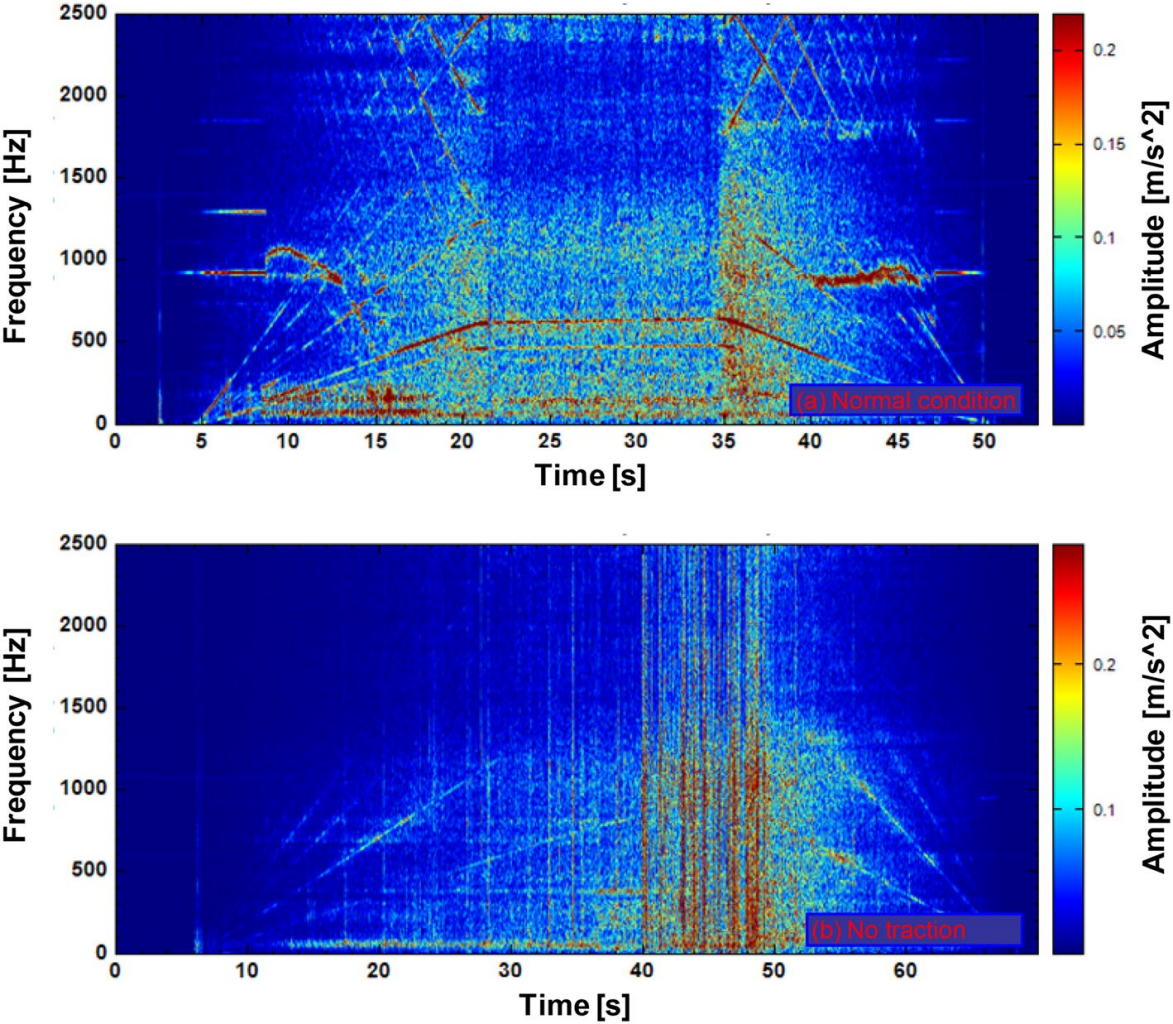
Pinion vertical vibration, (a) under normal condition, (b) with no traction.

The time–frequency diagram of the vertical vibration for the pinion is shown in Fig. 19. Figure 19a shows the whole process of the vehicle dynamics, accelerating first, then running at a constant speed and then decelerating. It is impressed that 628 Hz, i.e., meshing frequency, and 475 Hz, i.e., 12-times of the fundamental frequency of the rotor, both increased with the increase of the vehicle running speed during the traction acceleration process. And these two types of frequency remain unchanged during the constant speed process, and gradually decrease with the vehicle speed during the braking deceleration process. The vibration that nearly did not change with vehicle speed was mainly concentrated in the low frequency band.

## Conclusions

Traction drive system converts electrical energy into mechanical energy to drive the train, and adjusts the train speed and traction by changing the amplitude and frequency of AC voltage. In order to compare and analyze the influence of the electrical part of the traction drive system for vibration characteristics on vehicle components, an electromechanical coupling dynamic vehicle model was established in this work, which explicitly incorporated the electric-induced traction into transmission to study the influence of traction system on vehicle dynamics performance. The results indicate that transmission system could increase the vibration amplitude of the vehicle system, but it does not affect the frequency distribution of the vibration. The dynamics responses of the vertical, lateral and longitudinal acceleration on vehicle components, such as axle box and car-body were quantitative analyzed. Compared with the field test, it was found that 12-times of the fundamental frequency of the rotor always exists during the whole operation process of the train, such as traction, uniform speed and braking, and the frequency is transmitted to the bogie through the transmission system, causing high-frequency vibration. The vibration acceleration exhibited nearly little difference with or without traction force, especially at low frequency domain < 100 Hz.

## Appendix I

Vehicle parameters of the train and input conditions.

**Table:**

Name	Parameter
Gear ratio *a*	6.31
Gear transmission efficiency *η*_Gear_	0.98
Marshaling mode	Four motor vehicles and two trailers
Wheel diameter *D*, mm	846
Dead weight of motor vehicle $M_{{\text{m}}}$, t	35
Dead weight of trailer $M_{{\text{t}}}$, t	34
Intermediate DC voltage *U*_dc_, V	1500
Simulation step, s	0.0001
Basic resistance of train operation $W_{{\text{v}}}$, kN	$$\begin{gathered} W_{{\text{v}}} = \left[ {\left( {1.65 + 0.0247v} \right) \times M_{{\text{m}}} + \left( {0.78 + 0.0028v} \right) \times M_{{\text{t}}} } \right. \\ \left. {{ + }\left( {0.028 + 0.0078 \times \left( {N - 1} \right)} \right)v^{2} } \right] \times 9.80665 \times 10^{{{ - }3}} \\ \end{gathered}$$
Starting resistance of train, N/t	49

## Appendix II

The parameters of traction motor and control model.

**Table:**

Name	Parameter
Polar logarithm *n*_p_	2
Rated line voltage *U*, V	1050
Rated frequency *f*, Hz	60
Rated power *P*, kW	190
Rated speed *n*, rpm	1800
Maximum speed *n*_max_, rpm	4160
Stator resistance *R*_s_, Ω	0.111
Rotor resistance *R*_r_, Ω	0.099
Stator leakage inductance *L*_sσ_, mH	1.511
Rotor leakage inductance *L*_rσ_, mH	1.511
Mutual inductance *Lʹ*_m_, mH	45.03
Given flux linkage *Ψ*_s_*, Wb	2.75
Flux linkage tolerance Δ*Ψ*_s_, Wb	± 0.01
Torque tolerance Δ*T*_e_, Nm	± 10
Moment of inertia *J*, kg*m^2^	6
